# Co-receptor tropism and genetic characteristics of the V3 regions in variants of antiretroviral-naive HIV-1 infected subjects

**DOI:** 10.1017/S0950268819000700

**Published:** 2019-04-29

**Authors:** J. L. Guo, Y. Yan, J. F. Zhang, J. M. Ji, Z. J. Ge, R. Ge, X. F. Zhang, H. H. Wang, Z. W. Chen, J. Y. Luo

**Affiliations:** 1 Jiaxing Key Laboratory of Pathogenic Microbiology, Jiaxing Municipal Centre for Disease Control and Prevention, No.486, Wenqiao Road, Jiaxing, 314001, China; 2 Institute of AIDS Control and Prevention, Zhejiang Provincial Centre for Disease Control and Prevention, No.3399, Binsheng Road, Hangzhou, 310051, China

**Keywords:** Antiretroviral-naïve, co-receptor tropism, HIV-1, V3 region

## Abstract

Co-receptor tropism has been identified to correlate with HIV-1 transmission and the disease progression in patients. A molecular epidemiology investigation of co-receptor tropism is important for clinical practice and effective control of HIV-1. In this study, we investigated the co-receptor tropism on HIV-1 variants of 85 antiretroviral-naive patients with Geno2pheno algorithm at a false-positive rate of 10%. Our data showed that a majority of the subjects harboured the CCR5-tropic virus (81.2%, 69/85). No significant differences in gender, age, baseline CD4^+^ T-cell counts and transmission routes were observed between subjects infected with CXCR4-tropic or CCR5-tropic virus. The co-receptor tropism appeared to be associated with the virus genotype; a significantly more CXCR4-use was predicted in CRF01_AE infections whereas all CRF07_BC and CRF08_BC were predicted to use CCR5 co-receptor. Sequences analysis of V3 revealed a higher median net charge in the CXCR4 viruses over CCR5 viruses (4.0 *vs.* 3.0, *P* < 0.05). The predicted N-linked glycosylation site between amino acids 6 and 8 in the V3 region was conserved in CCR5 viruses, but not in CXCR4 viruses. Besides, variable crown motifs were observed in both CCR5 and CXCR4 viruses, of which the most prevalent motif GPGQ existed in both viral tropism and almost all genotypes identified in this study except subtype B. These findings may offer important implications for clinical practice and enhance our understanding of HIV-1 biology.

## Introduction

HIV remains a persistent problem for China and countries around the world since it was isolated from a culture derived from a lymph node biopsy sample of a patient with generalised lymphadenopathy in 1983 at the Institute Pasteur [1]. An estimated 36.9 (31.1–43.9) million people were living with HIV in the world according to the statistics from the Joint United Nations Programme on HIV/AIDS (UNAIDS) in 2017 [2]. HIV-1 can infect various immune cells such as CD4^**+**^ T cells, macrophages, monocytes and dendritic cells [3]. HIV-1 infection involves the interaction of the envelope glycoprotein gp120 with the CD4 molecule and also with chemokine co-receptors on the target cells. HIV-1 can be divided into CCR5 (R5), CXCR4 (X4) and dual/mixed (D/M) virus according to the co-receptors used for entry into host cells [4]. It is generally accepted that R5 viruses predominate in early infection stages and play a central role in transmission. While X4 or D/M viruses usually appear at late stages, associate with accelerated CD4^+^ T cells decline, increased risk of disease progression and death [5]. In 2007, a CCR5 antagonist named the maraviroc was approved by FDA for the HIV-1 treatment; it works by attaching to the CCR5 co-receptors on the cell surface to prevent R5 viruses from infecting the immune cells. The CCR5 antagonist is not recommended for people infected with X4 and D/M viruses [6]. In general, the investigation of HIV-1 co-receptor tropism has important significance due to its strong correlation with transmission, disease progression and the assessment of CCR5 antagonist for treatment [7].

Several countries have carried out the epidemiological study of the co-receptor tropism among antiretroviral-naive individuals and indicated that the prevalence of X4-tropic viruses fluctuated from 15% to 39.4% [8–12]. Recent reports of viral tropism in China mainly focused on male subjects or subjects infected with specific subtypes [13–15], which might not represent the general population, thus more comprehensive data on viral tropism of circulating HIV-1 strains of antiretroviral-naive populations in China are needed.

The third variable region (V3) of HIV-1 envelope protein is believed to be the principal determinant of co-receptor tropism [16]. Besides, due to its structural and conformational conservation, the V3 region might be a valuable target for vaccines for the induction of neutralising antibodies [17]. Our study, which focused on V3 sequence of the HIV-1 of the antiretroviral-naive patients living in Jiaxing, China, tried to predict the co-receptor tropism and monitor the disease progression, as well as propose prophylactic interventions for the effective control of HIV-1.

## Methods

### Ethical standards

The study was approved by the Review Board of the Ethics Committee of Jiaxing Municipal Centre for Disease Control and Prevention and was conducted according to the ethical requirements of the World Medical Association Declaration of Helsinki. Written informed consent was obtained from each study subject before the interview and test. The authors assert that all procedures contributing to this work comply with the ethical standards of the relevant national and institutional committees on human experimentation and with the Helsinki Declaration of 1975, as revised in 2008.

### Study population and sample collection

The subjects of the study were living in Jiaxing, an eastern coastal city of China with a resident population of 4.72 million by the end of 2018 according to the Jiaxing statistics bureau. From April 2015 to February 2016, newly reported HIV-1-positive individuals were recruited consecutively from the HIV surveillance network consisting of 119 sentinel laboratories affiliated to hospitals, health centres, blood centres and centres for disease control and prevention located in the Jiaxing city. A total of 99 subjects who were naive for antiretroviral therapy at the time of sampling were enrolled in our study. The epidemiology data including the transmission risk factors and demographic information (age, gender, etc.) were collected by trained interviewers. The venous blood samples were collected from the study subjects in the EDTA vacuum tube and CD4^+^ T-cell counts were measured within 24 h after sampling. Meanwhile, plasma samples were obtained by centrifugation and stored at −80 °C until further analysis was carried out.

### RNA extraction, amplification and sequencing

HIV-1 RNA was extracted from the plasma, and the partial envelope (env) genes (HXB2: 7002–7663) covering the entire V3 region were amplified by nested PCR with the outer primers 44F (forward, 5′-ACAGTRCARTGYACACATGG-3′) and 35R (reverse, 5′-CACTTCTCCAATTGTCCITCA-3′) and inner primers DR7m4 (forward, 5′-TGTAAAACGACGGCCAGTCTGTTAAATGGYAGYCTAGC-3′) and DR8m4 (reverse, 5′-CAGGAAACAGCTATGACCCTCCAATTGTYCCTCATAT-3′). Partial polymerase (pol) sequences (HXB2: 2147–3462) were amplified to determine the virus genotype as previously described [18]. Possible recombination was confirmed by bootscanning analysis using Simplot 3.5.1. (the corresponding amplification regions are labelled in the HIV-1 Gene Map for env and pol genes in the online Supplementary Fig. S1).

### Co-receptor usage prediction

The co-receptor usage of HIV-1 was determined by the V3 region amino acid sequence (bounded by C296 and C331 using HXB2 numbering). The Online tool Geno2pheno system was used to predict HIV-1 co-receptor usage available at https://coreceptor.geno2pheno.org/, with the false-positive rate (FPR) of 10%, which is in accordance with the current European guidelines [19]. The FPR indicates the probability of falsely classifying an R5 virus as X4. All FPR sequence prediction results >10% were considered as R5-tropic, whereas FPR ⩽ 10% were considered as X4-tropic.

### Genetic analysis of V3 region

V3 nucleotide sequences were aligned with Bioedit version 7.0 with minor manual adjustments and translated into amino acids. The net charge was calculated by subtracting negatively charged amino acids (aspartic acid (D) and glutamic acid (E)) from positively charged ones (arginine (R) and lysine (K)) in the V3 region, i.e. V3 net charge = (R + K)–(D + E). The potential N-linked glycosylation site (NGS, Asn-*X*-Thr/Ser, where *X* can be any amino acid except Pro) between amino acids 6 and 8 in the V3 region was analysed.

### Statistical analysis

Differences between groups were compared using the *χ*^2^ test for category variables and the Mann–Whitney *U* non-parametric test for continuous variables. Correlations of co-receptor usage with subject gender, transmission route and HIV-1 genotype were performed by Fisher's exact test. Correlations of co-receptor usage with age, CD4^+^ T-cell count and V3 net charge were performed by Mann–Whitney *U* non-parametric test. All analyses were conducted with SPSS software version 17.0 (SPSS Inc, Chicago, Illinois, USA). All tests were two-tailed and *P* < 0.05 was considered to be significant.

## Results

### Association between HIV-1 co-receptor tropism and patient's characteristics

**Fig. 1.**
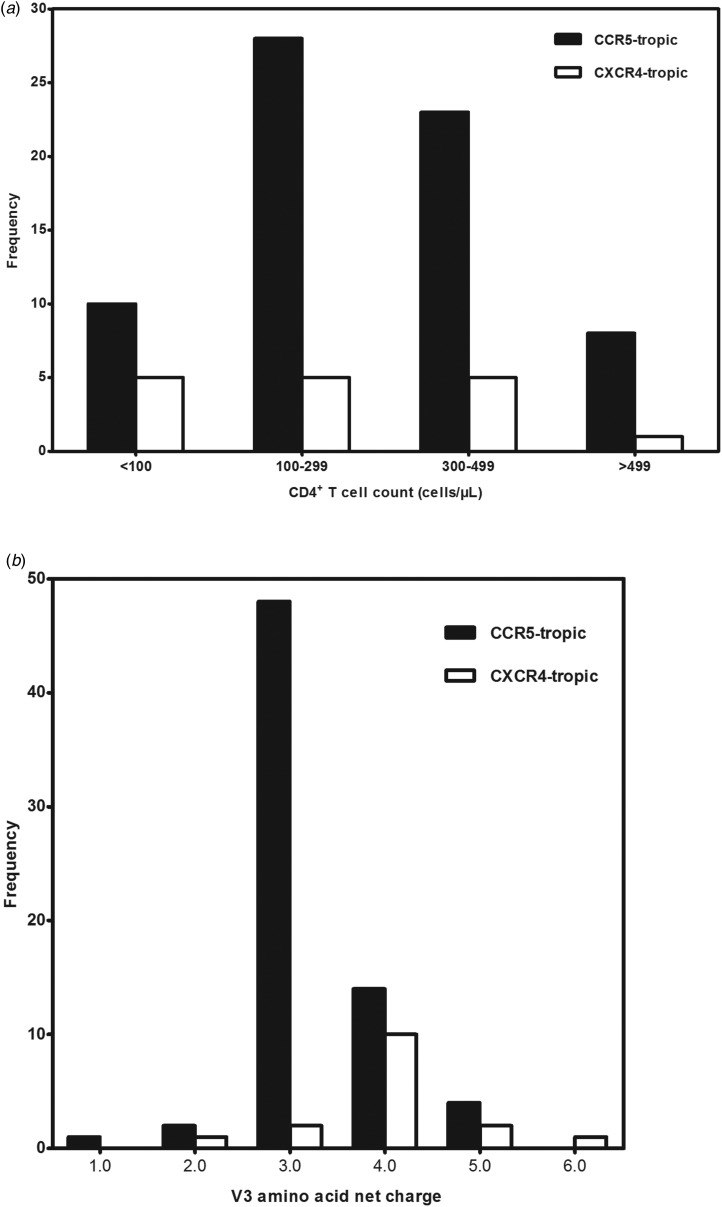
(a) Distribution of co-receptor tropism by CD4^+^ T-cell count. The *x*-axis represents the CD4^+^ T-cell count strata and *y*-axis represents the number of subjects in different CD4^+^ T-cell count strata. CCR5- and CXCR4-tropic strains are shown in black and white, respectively. (b) Histogram of V3 net charge distribution. The *x*-axis represents the individual net charge value and *y*-axis represents the counts for each discrete charge. CCR5- and CXCR4-tropic strains are shown in black and white, respectively.

A total of 99 antiretroviral-naive HIV-1 infected individuals living in Jiaxing were recruited in our study, 78.8% of the subjects were male. Median age was 35 years (IQR: 25.5–48.5). Eighty-eight subjects (88.9%) acquired the infection through sexual contact. Median CD4^+^ T-cell counts at sampling were 288 cells/μl (IQR: 190.0–379.0). Eighty-five envelope sequences were successfully amplified and sequenced from the plasma samples of the enrolled 99 subjects. With the FPR of 10%, the Geno2pheno system predicted the existence of X4-tropic viruses in 18.8% (16/85) of the subjects, whereas R5-tropic viruses in 81.2% (69/85) of the patients. The differences in age, gender and routes of transmission between subjects infected with X4-tropic or R5-tropic virus were compared and no statistical significance was discovered (Table 1). It is believed that CD4^+^ T-cell count is an important prognostic marker of disease progression; our results showed that R5-tropic and X4-tropic viruses were found at all CD4^+^ T-cell strata (Fig. 1a) and no significant difference was observed between the median CD4^+^ T-cell counts when comparing patients with X4-tropic viruses (257, IQR: 40.8–353.0) cells/μl *vs.* R5-tropic viruses (282, IQR: 187.0–367.0) cells/μl (*P* > 0.05) (Table 1).

**Table 1.** Characteristics of patients according to predicted tropism

	R5 (*n* = 69)	X4 (*n* = 16)	*P* value
Gender			
Male	53 (76.8%)	15 (93.8%)	0.175
Female	16 (23.2%)	1 (6.2%)	
Age, years			
Median (IQR)	35 (25.0–49.0)	38 (23.5–50.0)	0.991
CD4 count, cells/μl			
Median (IQR)	282 (187.0–367.0)	257 (40.8–353.0)	0.196
Transmission route			
MSM	22 (31.9%)	8 (50%)	0.217
Heterosexual	38 (55. 1%)	8 (50%)	
Others^a^	9 (13.0%)	0 (0%)	
Virus genotype (*n* = 85)			
CRF01_AE (*n* = 32, 37.6%)	20 (29.0%)	12 (75.0%)	0.001
Non CRF01_AE (*n* = 53, 62.4%)	49 (71.0%)	4 (25.0%)	
CRF07_BC	30 (43.5%)	0 (0%)	
CRF08_BC	14 (20.3%)	0 (0%)	
B	4 (5.8%)	1 (6.3%)	
Others^b^	1 (1.4%)	3 (18.7%)	

IQR, interquartile range; MSM, men who have sex with men; URF, unique recombinant forms.

Data are no. (%) of patients, unless otherwise indicated.

a Intravenous drug use and unknown.

b CRF55_01B and unique recombinant forms.

### Co-receptor usage in different genotypes

The genotypes of our study subjects (determined by pol genes) were distributed as follows: 32 (37.6%) CRF01_AE, 30 (35.3%) CRF07_BC, 14 (16.5%) CRF08_BC, five (5.9%) subtype B, two (2.4%) CRF55_01B and two (2.4%) URFs (unique recombinant forms). The distribution of HIV-1 genotypes and corresponding predicted co-receptors was shown in Table 1. The genotypes of the 16 X4-tropic viruses include 12 CRF01_AE, two CRF55_01B, one subtype B and one URF. Significantly more CXCR4-use was predicted in CRF01_AE infections, whereas all of the CRF07_BC and CRF08_BC were predicted to be R5-tropic.

### Co-receptor tropism prevalence in antiretroviral-naive patients differs regionally

In our study, the prevalence of X4 viruses in the subjects was 18.8% (16/85) and 37.5% (12/32) in CRF01_AE genotype. In order to gain a better understanding of the co-receptor tropism prevalence both in the study subjects and in CRF01_AE genotype, we reviewed recent related reports from domestic and international regions, as listed in Table 2. The prevalence of X4/DM viruses differs from study to study, with the percentages of CXCR4 use fluctuating between 2.0% and 39.4% in the study population, and ranging from 2.0% to 68.2% in CRF01_AE genotype. This discrepancy in the prevalence of co-receptor use in different studies might be due to the different patient populations, geographic region of viral acquisition, time from infection and methods used for co-receptor prediction. In our study, the prevalence of X4 virus in antiretroviral-naive HIV-1 patients (18.8%) was in keeping with other large sample size studies performed earlier in France, in Spain, in Canada and in Belgium [8–10, 11]. Besides, the prevalence of X4 virus in CRF01_AE infections (37.5%) was comparable to the investigations in Belgium, Shanghai and Hong Kong [11, 13, 15].

**Table 2.** Overview of prevalence of X4/dual mixed tropic (DM) viruses in antiretroviral-naive subjects reported in different studies

Source	Region	Subjects	Number^a^	Method	Prevalence of X4/DM virus	
					Study subjects (%)	CRF01_AE (%)
**Our data**	Jiaxing	HIV-1 positive	85	Genotypic	18.8	37.5
Li *et al*. [13]	Shanghai	MSM	276	Genotypic	26.1	40.9
Tsai HC *et al*. [14]	Taiwan	VCT male clients	108	Genotypic	26	–
Sabrina *et al*. [15]	Hong Kong	Subjects infected with B or CRF01_AE	203	Genotypic/phenotypic	–	39.1
Cui *et al*. [20]	Liaoning	MSM infected with CRF01_AE	59	Phenotypic	2.0	2.0
Li *et al*. [21]	Multicentre of China	CD4 < 350 cells/μl	201	Genotypic	36.3	68.2
Ghosn *et al*. [8]	France	Close to seroconversion	1387	Genotypic	15	–
SierraEnguita *et al*. [9]	Spain	Recent seroconverters	737	Genotypic	19.7	–
Brumme *et al*. [10]	Canada	HIV-1 positive	979	Phenotypic	18.2	–
Chalmet *et al*. [11]	Belgium	Recently diagnosed	539	Genotypic	19	39.5
Phuphuakrat *et al*. [12]	Thailand	HIV-1 positive	99	Genotypic	39.4	47.9

MSM, men who have sex with men; VCT, voluntary counselling and testing.

a Number of subjects that successfully passed the viral tropism test.

### Sequence characteristics of V3 regions

**Fig. 2.**
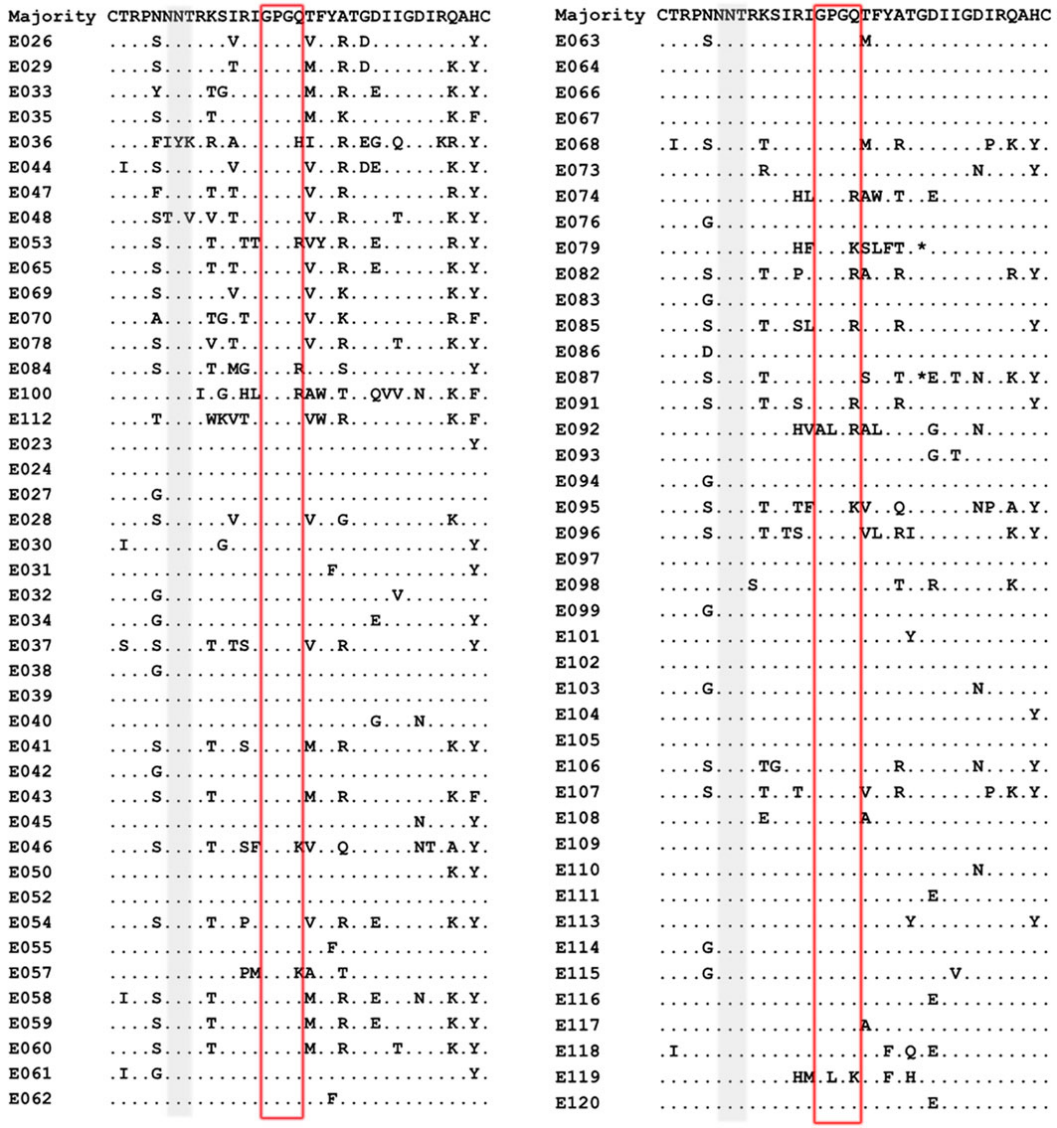
Comparison of V3 amino acid sequences from the viruses of patients studied. V3 sequences were aligned and translated into amino acids with Bioedit 7.0. The median number of amino acids for V3 was 35 (range 34–35). Dot (.) indicates identity with the majority sequence and asterisk (*) indicates a deletion mutation within the alignment. Positions in grey identify the predicted N-linked glycosylation site between positions 6 and 8 within the V3 region and the red box indicates the crown motif.

Our result showed that the X4-tropic viruses harboured a higher median net charge over R5-tropic viruses (+4.0 *vs.* + 3.0, *P* < 0.05). X4-tropic variants had a net charge between +2 and +6, with a majority of variants having the charge of +4, while R5-tropic viruses ranged between +1 and +5, with the highest frequency charge of +3 (Fig. 1b). The V3 sequences of the study subjects were aligned with Bioedit and translated into amino acids. The median number of amino acids for V3 was 35 (range 34–35). The sequon motif NNT was the only N-linked glycosylation pattern being observed in V3 region, all of the R5 viruses (69/69) and 87.5% of the X4 viruses (14/16) harboured the NNT glycosylation motif. Two X4 viruses lacking the N-linked glycosylation pattern had the amino acid motif IYK or TNV between amino acids 6 and 8 in the V3 region (Fig. 2). We also investigated the crown motif (amino acids 15–18) in the tip of V3 region, which was considered as the focal point of the potent neutralising antibody epitopes. Variable crown motifs were observed in both CCR5 and CXCR4 viruses, of which the most prevalent motif was GPGQ (83.5%), existed in both R5- and X4-tropic variants, and almost all genotypes except subtype B. In the V3 sequences, GPGR (8.2%), GPGK (4.7%), ALGR (1.2%), GLGK (1.2%) and GPGH (1.2%) were also observed. CRF07_BC and CRF08_BC harboured the only motif GPGQ, while variable crown motifs were observed in CRF01_AE including GPGQ, GPGR, GPGK, GLGK and GPGH. Subtype B was the only one that did not harbour the GPGQ in which only GPGR, GPGK, ALGR were observed (Table 3).

**Table 3.** Summary of variations in the crown motif of the V3 region according to co-receptor tropism and genotype

	Co-receptor tropism		Genotype				
	R5-tropic	X4-tropic	CRF01_AE	CRF07_BC	CRF08_BC	B	Others^a^
GPGQ (*n* = 71)	59	12	23	30	14	0	4
GPGR (*n* = 7)	4	3	4	0	0	3	0
GPGK (*n* = 4)	4	0	3	0	0	1	0
GLGK (*n* = 1)	1	0	0	0	0	1	0
ALGR (*n* = 1)	1	0	1	0	0	0	0
GPGH (*n* = 1)	0	1	1	0	0	0	0

a CRF55_01B and unique recombinant forms.

## Discussion

HIV infection is a chronic viral infection and usually a life-long antiretroviral therapy is required. However, a great number of patients in the world have developed drug resistance to current antiretroviral drugs such as reverse transcriptase inhibitors and protease inhibitors [22]. Advent of the new drug CCR5 antagonists is a crucial step in the fight against HIV-1, while viral tropism has to be determined before initiating treatment with the drug. Here, we adopted a now generally accepted genotypic method Geno2pheno to define viral tropism [19]. Tropism was determined in 85 samples (86.0%) and could not be determined in 14 samples (14.0%) because of the great diversity of the HIV-1 envelope genes and the low viral loads in these subjects. Among the 85 subjects that acquired the env sequences, no statistical significance of differences in age, gender, routes of transmission between subjects infected with X4-tropic or R5-tropic virus was observed, which was in agreement with all related representative studies we searched [8, 9, 10, 11]. However, the differences in baseline CD4^+^ T-cell counts between the two groups remain controversial in different studies. No significant difference of baseline CD4^+^ T-cell counts between the two groups was observed in our study, which was in agreement with some previous studies [8, 9]. While, other studies showed that patients with X4 viruses harboured significant lower baseline CD4^+^ T-cell counts than those with R5 viruses [10, 11]. As is known, blood CD4^+^ T-cell counts are dynamic with a transient reduction followed by recovery to near-normal concentrations and then slowly decrease in untreated infections [3]. X4 viruses frequently emerge at high CD4^+^ T-cell counts, and then slowly decrease if untreated [23]. The different intervals between the emergence time of X4 viruses and the enrolment time for CD4^+^ T-cell counts may help explain the discrepant results of differences of baseline CD4^+^ T-cell counts between subjects infected with X4-tropic or R5-tropic virus in various studies.

Our data showed that a majority of viruses (81.2%, 69/85) were predicted to be R5-tropic, indicating that CCR5 antagonists would still be promising drugs for the treatment of HIV-1 in the future. Besides, 18.8% (16/85) of the HIV-1 variants could be predicted as X4-tropic, which was comparable to some larger studies performed in West Europe or North America (Table 3). Evidence suggested that X4-tropic viruses might be more virulent and associated with increased risk of disease progression and death [24, 25]. A study in Spain suggested the X4-tropic viruses were increasing over time and almost doubled from 1997 to 2012 in new HIV-1 infections [9], which alert us to strengthen the viral tropism monitoring among newly diagnosed HIV-1 infections to provide optimised therapy and improve clinical outcome.

Previous reports showed that different HIV genotypes may have specific co-receptor preferences [26]. Here we found that all CRF07_BC and CRF08_BC strains were predicted to use CCR5 co-receptor. Besides, significantly more CXCR4-use was predicted in CRF01_AE infections. At present, sexual transmission is the primary mode of transmission in China and CRF01_AE has become the predominant subtype in Chinese HIV-1 sexually infected patients [21], the high CXCR4-use in CRF01_AE may lead to reduced susceptibility to CCR5 antagonists, which emphasise the need for viral tropism screening in CRF01_AE infections in China.

Evidence suggests that a higher net charge and loss of the NGS (amino acids 6–8) in the V3 region are associated with CXCR4 use [27, 28]. Our result showed that the net charge of X4 viruses (median 4.0) was higher than that of R5 viruses (median 3.0) (*P* < 0.05). We found that 12.5% of the X4 variants lacked the NGS, while the NGS was conserved in the R5 viruses. It is speculated that the highly conserved NGS in R5 viruses may help increase transmission efficiency in the early stage of HIV-1 infection by blocking antibodies in the host immune system and enhancing binding to CCR5 co-receptor [29].

It is reported that crown motifs of the V3 region vary with subtypes and co-receptor tropism. For instance, subtype C usually harbours GPGQ in R5 viruses and GPGR in X4 viruses, whereas the subtype B generally contains a GPGR motif irrespective of co-receptor usage [30, 31]. Our study revealed a variable crown motif in both R5- and X4-tropic viruses. The crown motifs such as GPGQ, GPGR, GPGK, GLGK and GPGH were observed in CRF01-AE irrespective of co-receptor usage. While all the CRF07_BC and CRF08_BC were R5 viruses with the only motif GPGQ. The crown motifs predicted on the most common variants of the tip of the V3 region may provide important implications for HIV peptide vaccine design.

However, a major limitation of the study is our small sample size, so data should be interpreted cautiously to warrant population-level inferences, a larger molecular epidemiology of co-receptor usage prevalence would be required to strengthen these findings. Besides, our sequencing method only detects the predominant variants, the prevalence of X4 viruses may be underestimated if they are present as minor populations of quasispecies. In general, our study provides an insight of the co-receptor tropism prevalence among antiretroviral-naive patients living in Jiaxing, which may help monitor disease progression and optimise therapy regimens, as well as propose prophylactic interventions for effective control of HIV-1.

## Supplementary material

For supplementary material accompanying this paper visit https://doi.org/10.1017/S0950268819000700. click here to view supplementary material
