# Supplementary material for: Co-receptor tropism and genetic characteristics of the V3 regions in variants of antiretroviral-naive HIV-1 infected subjects
Source: Epidemiol Infect. 2019 Apr 29;147:e181. doi: 10.1017/S0950268819000700 (PMC6518647; doi:10.1017/S0950268819000700)

**Supplementary Material for**

**Co-receptor tropism and genetic characteristics of the V3 regions in variants of** **antiretroviral-naive HIV-1 infected subjects**

JL. Guo^1^*, Y.Yan^1^*, JF. Zhang^2^*, JM. Ji^1^, ZJ. Ge^1^, R. Ge^1^, XF. Zhang^1^, HH. Wang^1^, ZW. Chen^1^, JY. Luo^1^†

1. Jiaxing Key Laboratory of Pathogenic Microbiology, Jiaxing Municipal Centre for Disease control and Prevention. No.486, Wenqiao Road, Jiaxing, PR China, 314001.

2. Institute of AIDS Control and Prevention, Zhejiang Provincial Centre for Disease Control and Prevention, No.3399, Binsheng Road, Hangzhou, PR China, 310051.

*These authors contributed equally in this study

†Correspondence footnote:

Jianyong Luo, Jiaxing Municipal Centre for Disease Control and Prevention. No.486, Wenqiao Road, Jiaxing, PR China, 314001.

Tel: 86-0573-83685311, Fax: 86-0573-83685811, E-mail: luojianyong1116@sina.com

**Figure Legend**

**Supplementary Figure S1. Diagram of HIV-1(HXB2) genome structure.**

The full-length genome of HIV-1 (HXB2) was drawn with the online tool in the Los Alamos HIV sequence database (<https://www.hiv.lanl.gov/content/sequence/DRAW_CRF/recom_mapper.html>) and shown in the Figure S1. The corresponding amplification regions in our study for partial envelope gene (HXB2: 7002-7663) and partial polymerase gene (HXB2: 2147-3462) were marked with red and blue, respectively.


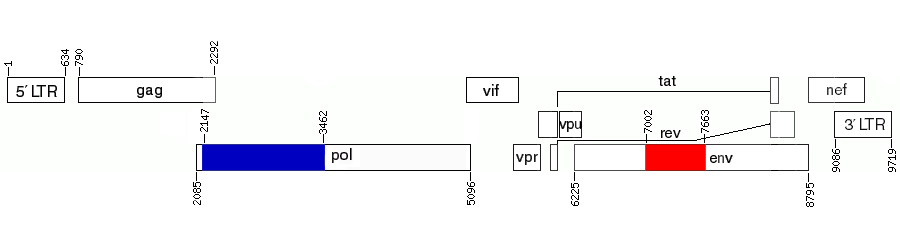

Supplement: Supplementary file 1 [file S0950268819000700sup001.docx]
